# Arsenic level in toenails is associated with hearing loss in humans

**DOI:** 10.1371/journal.pone.0198743

**Published:** 2018-07-05

**Authors:** Xiang Li, Nobutaka Ohgami, Ichiro Yajima, Huadong Xu, Machiko Iida, Reina Oshino, Hiromasa Ninomiya, Dandan Shen, Nazmul Ahsan, Anwarul Azim Akhand, Masashi Kato

**Affiliations:** 1 Department of Occupational and Environmental Health, Nagoya University Graduate School of Medicine, Nagoya, Japan; 2 Voluntary Body for International Health Care in Universities, Nagoya, Japan; 3 Department of Genetic Engineering and Biotechnology, University of Dhaka, Bangladesh; Stony Brook University, Graduate Program in Public Health, UNITED STATES

## Abstract

Arsenic (As) pollution in drinking water is a worldwide health risk for humans. We previously showed hearing loss in young people who live in areas of As-polluted drinking water and in young mice orally treated with As. In this study, we epidemiologically examined associations between As levels in toenails and hearing in 145 Bangladeshi aged 12–55 years in 2014. Levels of As in toenails, but not those in urine, were shown to be significantly correlated with hearing loss at 4 kHz [odds ratio (OR) = 4.27; 95% confidence interval (CI): 1.51, 12.05], 8 kHz (OR = 3.91; 95% CI: 1.47, 10.38) and 12 kHz (OR = 4.15; 95% CI: 1.55, 11.09) by multivariate analysis with adjustments for age, sex, smoking and BMI. Our experimental study further showed a significant association between As levels in inner ears and nails (r = 0.8113, p = 0.0014) in mice orally exposed to As, suggesting that As level in nails is a suitable index to assess As level in inner ears. Taken together, the results of our study suggest that As level in nails could be a convenient and non-invasive biomarker for As-mediated hearing loss in humans.

## Introduction

Exposure to arsenic (As) via drinking water is a health risk for humans [1–3]. Previous studies showed that exposure of humans to As was associated with cancer of the bladder, kidney, skin, prostate, lung and liver [4] and with cardiovascular disease [5]. It was also shown that exposure to As was associated with neurological diseases including cognitive impairment in children [6, 7] and adults [8] and with diabetes mellitus [9–12].

Levels of toxic elements in noninvasive biological samples including nails, hair and urine have been determined to investigate associations with hearing loss in humans [13, 14]. A univariate analysis showed a significant correlation of As levels in fingernails with amplitude of distortion product otoacoustic emission (DPOAE) at 2 kHz, which reflects activity levels of outer hair cells, in 30 subjects aged 9–78 years residing in the gold mining community of Bonanza, Nicaragua [15]. Thus, it is possible that As in nails is a reliable index for As-mediated hearing loss in humans. However, there is no direct evidence showing correlations between As levels in toenails and hearing levels.

In our previous study using pure tone audiometry (PTA), an association of oral exposure to As via drinking water with hearing loss in young people was shown by multivariate analysis with adjustments for confounders including age, smoking, sex and BMI [16]. However, multivariate analysis has not been performed to demonstrate the association of As levels in nails with hearing loss in humans, although age and smoking are known to be strong factors affecting hearing levels in humans [14, 17–19].

In experimental studies, oral exposure to As has been shown to cause several health risks including carcinogenesis [20] and cardiovascular diseases [21]. Levels of toxic elements in inner ears, which are known to be the sensory organ for hearing, have been determined to demonstrate the correlation with hearing loss [22, 23]. Exposure of guniea pigs to As at 200 mg/L via intraperitoneal injection for 2 months resulted in morphological impairments of the inner ear [24]. Our previous study has showed that exposure of young mice to As via drinking water caused accumulation of As in inner ears, resulting in hearing loss [16]. However, it is not clear whether there is a correlation between As levels in inner ears and nails in mice.

In this study, we epidemiologically and experimentally investigated whether As levels in nails are reliable as non-invasive indexes for As-mediated hearing loss in humans and whether As levels in nails are associated with As levels in inner ears in mice.

## Materials and methods

### Epidemiological study

We obtained information on age, sex, smoking history, weight and height in 145 healthy subjects aged from 12 to 55 years by self-reported questionnaires in Bangladesh as previously described [14]. We obtained informed consent in written form from all of the subjects. For subjects aged 12 to 18 years, we obtained consent in written form from their parents. The subjects were only Bangladeshi people who lived mainly in rural areas. The subjects living in one area drink tap water and the rest of the subjects living in another area drink tube well water contaminated with As. The subjects included students, housewives and businessmen. The subjects did not have portable music players with earphones and had no occupational exposure to wielding fumes. None of the subjects had a habit of drinking alcohol. We excluded only subjects who had a history of ear diseases or illness at the time of the survey. We calculated body mass index (BMI) by diving weight (kg) by the square of height (m^2^) and used definitions of underweight (< 18.5 kg/m^2^), normal weight (18.5–24.9 kg/m^2^) and overweight (≥ 25 kg/m^2^) set by the WHO [25]. We set the mean value of age (29.6 years) of the subjects in this study as the cut-off value for age. We collected 0.1–2 cm samples of toenails from each subject by using a ceramic nail clipper. We also collected urine samples from each subject and stored the samples in 50 ml sterile tubes at -80°C until measurements. We determined the hearing levels at 1, 4 and 8 kHz in addition to 12 kHz of the participants by PTA, since hearing level at 12 kHz was shown to be sensitive to environmental stresses [14, 17, 26]. We used an iPod with earphone-type headphones (Panasonic RP-HJE150) in a soundproof room as described previously [27, 28]. We output pure tones at each frequency to a subject until the subject recognized the sound. We stood behind the subject and provided the subject with an initial stimulus of 5 decibels (dB) and then increased the sound level by 5 dB step-by-step. The subject raised their hand when they recognized the sound. We evaluated the sound level recognized by each subjects as hearing threshold. Pure tones at each frequency from the earphones in the soundproof room were verified by using a noise level meter (Type 6224 with an FFT analyzer, ACO CO., LTD, Japan). The epidemiological study was approved by Nagoya University International Bioethics Committee following the regulations of the Japanese government (approval number: 2013–0070) and the Faculty of Biological Science, University of Dhaka (Ref. no. 5509/Bio.Sc).

### Experimental study

Hairless mice having the C57BL/6J background (1 month old, female, body weight of 10–15 g) were procured from Hoshino Laboratory Animal, Inc. Three or four mice were housed in a cage under super pathogen-free (SPF) conditions with a standard temperature of 23 ± 2°C and a 12-h light/dark cycle. The mice were fed a standard rodent chow (Clea Rodent Diet CE-2). Neither randomization nor blinding investigation were used in this animal study. In brief, we exposed mice (n = 7) to 22.5 mg/L of sodium arsenite (NaAsO_2_, Sigma-Aldrich) dissolved in distilled water for 2 months via drinking water and changed the drinking water every week as previously described [16]. The exposure dose was based on the As exposure for mice at 10 and 100 ppm via drinking water in a previous study [29]. The control group (n = 6) was given just distilled water. After exposure for 2 months, mice were anesthetized with isoflurane and sacrificed by decapitation. Nails and inner ears were collected and kept in a 1.5 ml tube. For the collection of inner ears, we first identified temporal bones at the bottom of the skull and then carefully removed the cranial nerves and tissues using standard forceps. The inner ears were dislodged by pushing down on the posterior semicircular canal with the thumb of a hand and fixing the tip region of the otic bone capsule with standard forceps. After carefully removing extra tissues adhered to the inner ears, we used the inner ears for ashing. The experimental study was approved by the Institutional Animal Care and Use Committee in Nagoya University (approval number: 28251) and followed the Japanese Government Regulations for Animal Experiments.

### Measurement of As levels in biological samples

As levels in biological samples including toenails and urine were measured by inductively coupled plasma mass spectrometry (ICP-MS; Agilent 7500cx) as described previously [14, 22, 23]. In brief, toenails were washed with detergent water. One or two drops of acetone were added and then the samples were kept at room temperature until starting the ashing. Samples were ashed by incubation in 3 ml of HNO_3_ at room temperature overnight followed by incubation at 80°C for 3 hours. Samples were further incubated in 1–1.5 ml of H_2_O_2_ at 80°C for 3 hours. After cooling, Milli-Q water was added to the samples to adjust the final volume to 5 ml. In the case of urine, 4 ml of urine was incubated in 1 ml of HNO_3_ at room temperature overnight followed by incubation at 80°C for 24–72 hours. After cooling, the ashed urine samples were centrifuged at 2,000 rpm (= 269 g) for 1 min and Milli-Q water was added to the samples to adjust the final volume to 5 ml. Levels of As in urine were normalized by specific gravity using the following formula: SG-corrected concentration = raw hormone concentration × [(SG_target_− 1.0)/(SG_sample_—1)], where SG_target_ is a population mean SG [30]. In this study, the mean SG was 1.012 for the subjects. In the case of murine samples, nails were rinsed with Milli-Q water 3 times and air-dried at room temperature. Ashing of the samples was then performed with the same protocol as that described above. Levels of As in biological samples were determined by ICP-MS.

### Statistical analysis for epidemiological study

All statistical analyses were performed by JMP software (version 11.0.0). In univariate analysis, the Mann-Whitney *U* test and Steel-Dwass test were used to detect significant differences in hearing levels between the groups because the hearing levels were discontinuous variables. In multivariate analysis, binary logistic regression analysis was performed with adjustments for age, sex, smoking and BMI as confounding factors. We used the method in a previous study [26] to categorize subjects with an auditory threshold higher than the cut-off value at each frequency as hearing loss. We set hearing thresholds [1 kHz (≥ 7 dB), 4 kHz (≥ 10 dB), 8 kHz (≥ 24 dB), and 12 kHz (≥ 45 dB)] with the mean values of hearing levels at each frequency to divide the subjects into two groups. In this study, values of p < 0.05 were considered statistically significant.

### Statistical analysis for experimental study

In our experimental study, Spearman’s correlation coefficients were used to evaluate the association between As levels in inner ears and those in nails. Values of p < 0.05 were considered statistically significant.

## Results

### Characteristics of the study participants

Table 1 shows the characteristics of the subjects, which were described in our previous report [14]. The hearing thresholds in the older group (≥ 29.6 years old, n = 68) were significantly higher than those in the younger group (< 29.6 years old, n = 77) at 1 kHz (p = 0.0098), 4 kHz, 8 kHz and 12 kHz (p < 0.0001) (Table 2). The hearing thresholds in females (n = 76) were significantly higher than those in males (n = 69) at 4 kHz (p = 0.0257), 8 kHz (p = 0.0004) and 12 kHz (p = 0.0066) (Table 2). No significant differences were found in hearing thresholds among the three BMI groups (Table 2). Smokers (n = 31) had higher hearing thresholds than those in non-smokers (n = 114) at 1 kHz (p = 0.0057), 4 kHz (p < 0.0001), 8 kHz (p = 0.0002) and 12 kHz (p < 0.0001) (Table 2).

**Table 1:** Characteristics of the study participants.

Characteristics	mean ± SD	Variables	Participants [n (%)]
Age	29.6 ± 11.0	< 29.6	77
		≥ 29.6	68
Sex		Male	69
		Female	76
Smoking history		No	114
		Yes	31
BMI	22.0 ± 3.4	< 18.5	23
		18.5 ≤ BMI < 25	94
		≥ 25	28

**Table 2:** Associations between hearing levels and confounding factors including age, BMI, sex and smoking.

	Hearing levels (mean ± SD)			
variables	1 kHz	4 kHz	8 kHz	12 kHz
Age				
< 29.6	6.10 ± 3.50	7.01 ± 4.39	19.29 ± 9.79	35.39 ± 15.64
≥ 29.6	7.43 ± 4.69	13.31 ± 9.60	29.93 ± 12.53	56.69 ± 22.15
p value	0.0098	p < 0.0001	p < 0.0001	p < 0.0001
Sex				
Male	6.59 ± 3.88	8.84 ± 6.81	21.23 ± 12.08	40.94 ± 21.61
Female	6.84 ± 4.38	10.99 ± 8.76	27.04 ± 11.98	49.41 ± 21.13
p value	0.7697	0.0257	0.0004	0.0066
Smoking				
No	6.40 ± 3.85	8.42 ± 5.70	22.06 ± 10.56	40.70 ± 18.38
Yes	7.90 ± 4.96	15.65 ± 11.74	32.42 ± 14.94	62.58 ± 24.49
p value	0.0057	p < 0.0001	0.0002	p < 0.0001
BMI				
< 18.5	6.52 ± 4.38	7.17 ± 3.64	19.78 ± 5.93	42.17 ± 16.91
18.5 ≤ BMI < 25	6.49 ± 3.35	10.05 ± 6.66	24.26 ± 12.11	44.95 ± 21.61
≥ 25	7.68 ± 6.01	11.96 ± 12.72	28.04 ± 15.65	49.46 ± 25.40
p value	0.6148	0.1797	0.1346	0.6595

### Association between As levels in biological samples and hearing levels by univariate analysis

**Fig 1:**
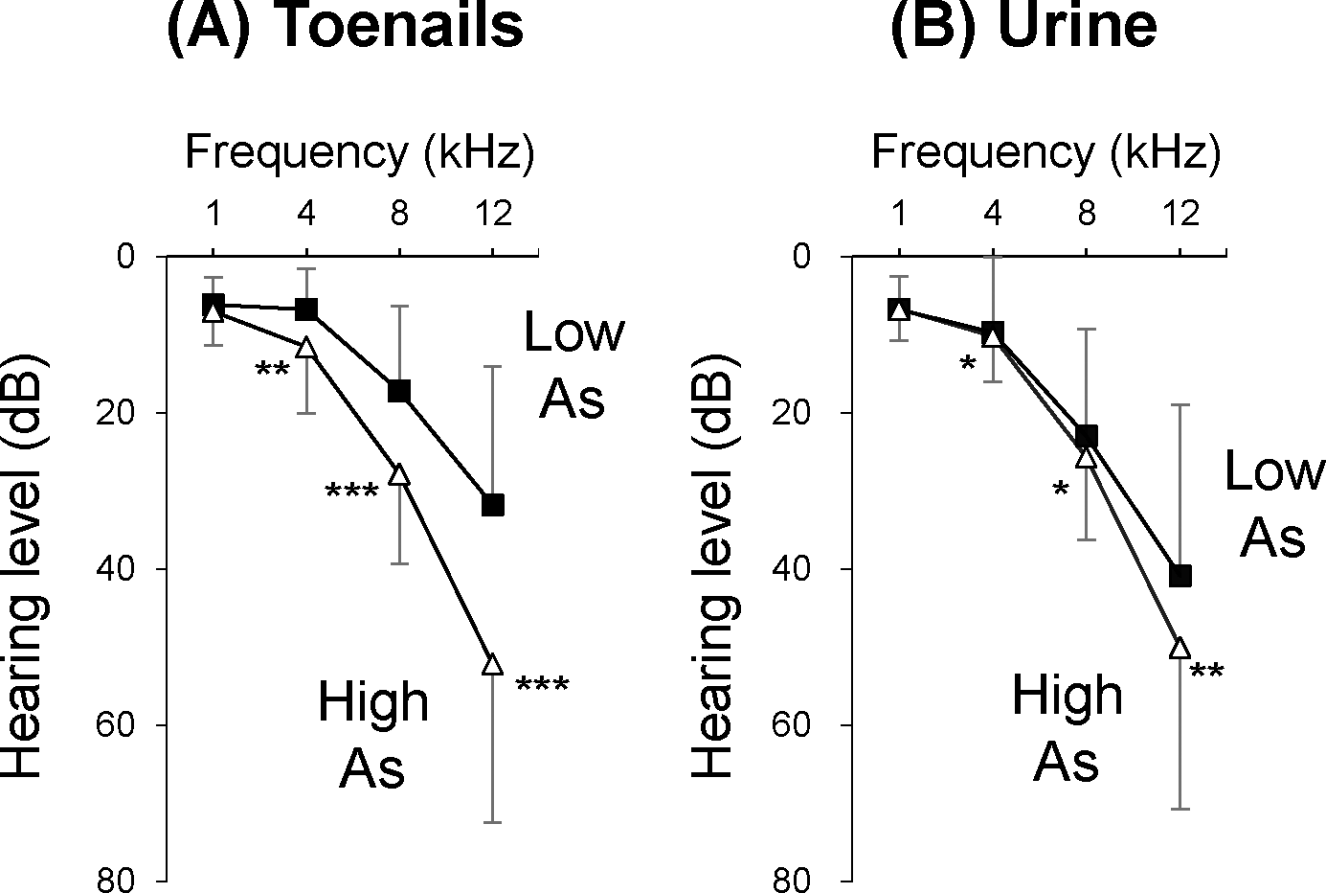
Association between hearing thresholds and As levels in biological samples in humans. (A) Hearing levels (mean ± SD) at 1, 4, 8 and 12 kHz in the high As group (≥ 0.60 μg/g; n = 97) and low As group (< 0.60 μg/g; n = 48) in toenails are presented. (B) Hearing levels (mean ± SD) at 1, 4, 8 and 12 kHz in the high As group (≥ 76.12 μg/L; n = 73) and low As group (< 76.12 μg/L; n = 72) in urine samples are presented. Significant differences (*p<0.05; **p < 0.01; ***p<0.001) were determined by the Mann-Whitney *U* test.

Concentrations of As (mean ± SD) in toenails and urine were 1.38 ± 1.17 μg/g and 90.27 ± 103.04 μg/L, respectively (Table 3). In this study, we set the cut-off values based on the receiver operating characteristic (ROC) curve and the highest Youden index [31]. We categorized the subjects into two groups at 0.60 μg/g in toenails and 76.12 μg/L in urine (Table 3). The mean ages of subjects in the high and low As groups were 31 years and 27 years, respectively. We found that hearing thresholds were significantly higher in the high As group in toenails (n = 97) at 4 kHz (mean = 10.48 dB; p = 0.0023), 8 kHz (mean = 26.71 dB; p < 0.0001) and 12 kHz (mean = 51.64 dB; p < 0.0001) than those in the low As group (n = 48) at 4 kHz (mean = 9.44 dB), 8 kHz (mean = 21.81 dB) and 12 kHz (mean = 39.03 dB) (Fig 1A). We also found that the group with high As levels in urine (n = 73) had significantly higher hearing thresholds at 4 kHz (mean = 10.41 dB; p = 0.0200), 8 kHz (mean = 25.96 dB; p = 0.0104) and 12 kHz (mean = 50.07 dB; p = 0.0015) than those in the low As group (n = 72) at 4 kHz (mean = 9.51 dB), 8 kHz (mean = 22.57 dB) and 12 kHz (mean = 40.63 dB) (Fig 1B).

**Table 3:** As levels in biological samples from humans.

	Mean ± SD	Range	Cut-off value
Toenails (μg/g)	1.38 ± 1.17	0.06–6.22	0.60
Urine (μg/L)	90.27 ± 103.04	4.17–1013.5	76.12

### Association between hearing thresholds and As levels in biological samples

We next performed logistic regression analysis with adjustments for age, sex, smoking history and BMI to determine the risk of hearing loss in subjects with high As in biological samples (Table 4). In this study, we followed the method used in a previous study [16] to define subjects with hearing loss as subjects with hearing thresholds more than the cut-off values at each frequency. Levels of As in toenails were significantly associated with hearing loss at 4 kHz [odds ratio (OR) = 4.27; 95% confidence interval (CI): 1.56, 12.70], 8 kHz (OR = 3.91; 95% CI: 1.50, 10.77) and 12 kHz (OR = 5.58; 95% CI: 2.21, 15.07) (Table 4). No significant correlations were found between As levels in urine and hearing loss at any frequency (Table 4). We further shifted the cut-off values of the independent variables dichotomizing As levels in biological samples to verify the models. We found that the significance of ORs in toenails remained when the cut-off values in toenails were shifted from 0.30 to 0.70 μg/g, while As levels in urinary samples did not show significant ORs even when the cut-off values were shifted from 10 to 380 μg/L.

**Table 4:** Adjusted ORs (95% CI) for hearing loss and As levels in biological samples (n = 145)^a^.

	1 kHz (≥ 7 dB)	4 kHz (≥ 10 dB)	8 kHz (≥ 24 dB)	12 kHz (≥ 45 dB)
**As in toenails**				
Low	Reference	Reference	Reference	Reference
High	1.28 (0.43–3.83)	4.27** (1.51–12.05)	3.91** (1.47–10.38)	4.15** (1.55–11.09)
**As in urine**				
Low	Reference	Reference	Reference	Reference
High	0.69 (0.29–1.63)	1.89 (0.83–4.29)	1.48 (0.65–3.34)	1.15 (0.52–2.56)

OR, odds ratio; CI, confidence interval. References mean 1.00.

^a^Adjusted for age, sex, smoking history and BMI.

**p < 0.01.

### Mice orally exposed to As showed an association between As levels in inner ear and nails

**Fig 2:**
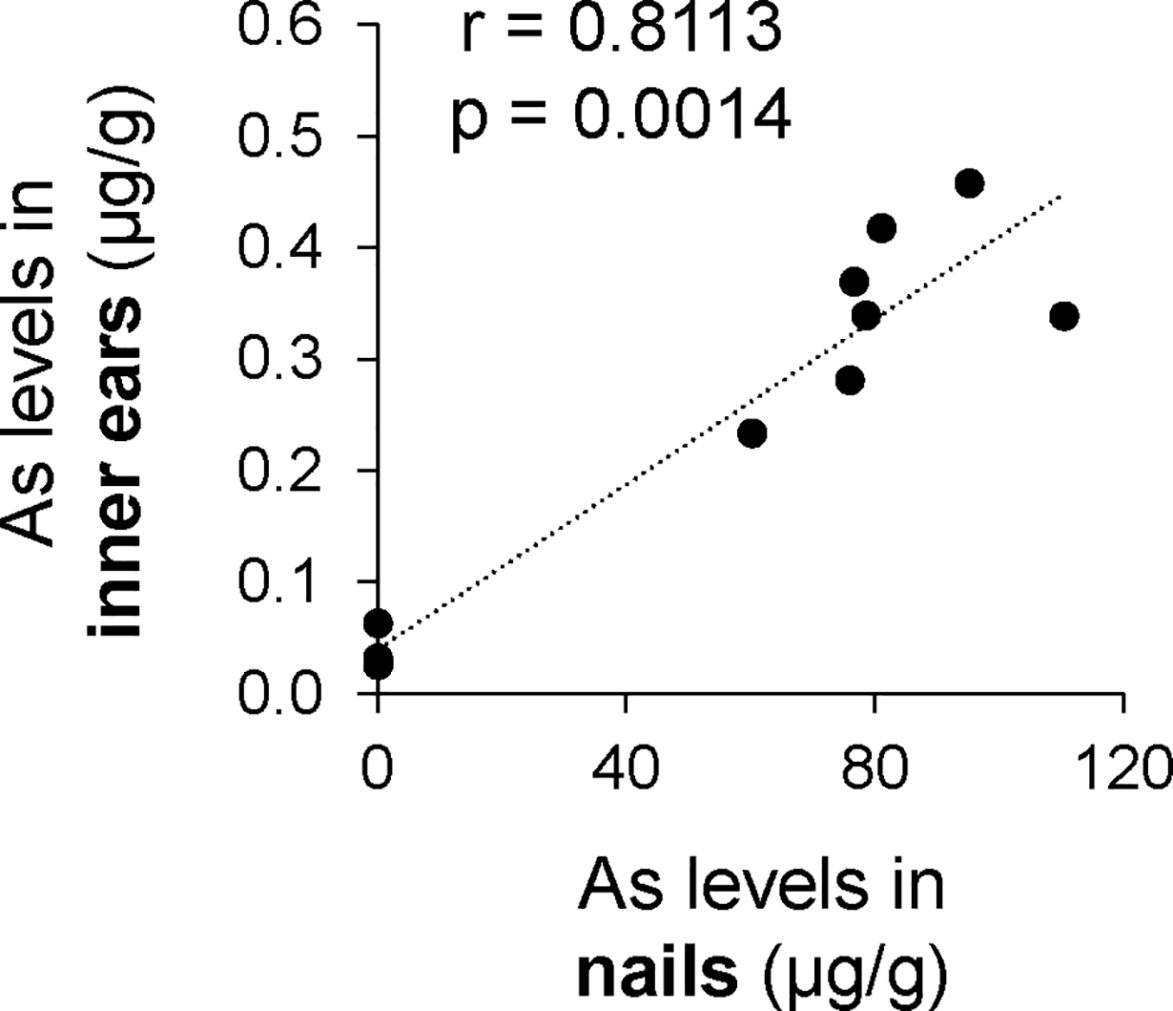
Correlation between As levels in inner ear and nails in mice. Correlation between As levels in inner ears and nails was determined by Spearman correlation coefficients.

We finally performed an experimental study to determine the correlation between As levels in nails and inner ears in mice. After exposure of mice to As via drinking water for 2 months, we measured As levels in nails and inner ears from the exposed group and the control group. We found that there was a significant correlation (r = 0.8113, p = 0.0014) between As levels in inner ears and nails (Fig 2).

## Discussion

### As level in toenails is a possible biomarker associated with hearing loss

In our epidemiological study, As levels in toenails were shown to be significantly correlated with hearing loss at 4, 8 and 12 kHz in humans by multivariate analysis with adjustments for age, sex, smoking and BMI. Our experimental study also showed that As level in nails is a reliable index to assess As level in inner ears. Thus, the results of our combined study suggest that As level in toenails is a possible biomarker associated with hearing loss at 4, 8 and 12 kHz in humans.

### Association between chronic exposure to As and hearing loss

The levels of an element in nails and hair are indexes reflecting chronic exposure status, while those in blood and urine samples are regarded as indexes of acute exposure [32, 33]. In our multivariate analysis, As levels in urine were not significantly correlated with hearing loss. Correspondingly, previous studies in which multivariate analyses with adjustments for age, sex and smoking were performed showed that As levels in urine were not associated with hearing loss [34–36]. Therefore, our results suggest that chronic exposure to As, but not acute exposure, is required for hearing loss in humans. In this study, we determined associations between As levels in biological samples and hearing levels in Bangladeshi only. Previous studies showed that races are associated with hearing levels [37, 38]. Thus, further study is needed to verify the associations between As levels in toenails and hearing loss in other countries.

### Association between As levels in hair and hearing loss at 12 kHz

The association between As levels in hair and hearing loss in humans aged 12–55 years was also analyzed in this study (S1 Table). As levels in hair were significantly associated with hearing loss at 12 kHz (OR = 2.94; 95% CI: 1.20, 7.20) but not with hearing loss at 4 or 8 kHz (S1 Table). Thus, our multivariate analysis showed that the significant association of As levels in hair with hearing loss was limited to an extra-high frequency (12 kHz), which is not necessary for daily communication in humans. A previous study showed that children living in an As-polluted area had hearing losses at 125, 250, 500, 1,000 and 8,000 Hz [39]. The As level in hair was 3.26 μg/g (mean value) in the previous study, while the As level in hair was 0.50 μg/g (mean value) in this study, about 6.5-times lower than that in the previous study. Therefore, it is possible that different levels of As in hair are associated with hearing loss at different frequencies.

### Relative contributions of As in toenails and confounders to hearing loss

Age and smoking are known to be strong confounders for hearing loss. In this study, multivariate analysis showed that age was significantly associated with hearing loss at 4 kHz (OR = 4.25; 95% CI: 1.83, 9.89), 8 kHz (OR = 4.37; 95% CI: 1.84, 10.39) and 12 kHz (OR = 3.31; 95% CI: 1.46, 14.51). Smoking also had significant associations with hearing loss at 4 kHz (OR = 3.78; 95% CI: 1.17, 12.15) and 12 kHz (OR = 4.49; 95% CI: 1.39, 14.51). We then used the McFadden’s Pseudo *R*^*2*^ values [40] to determine the relative contributions (%) of As in toenails and the other confounders including age to hearing loss at each frequency in the multivariate analysis (S2 Table). The relative contribution of As in toenails (17.33%) to hearing loss at 12 kHz was higher than the relative contributions of age (16.53%) and smoking (13.78%), while the relative contributions of age to hearing loss (20.75% at 4 kHz and 20.41% at 8 kHz) were higher than those of As in toenails (14.54% at 4 kHz and 13.70% at 8 kHz), smoking (9.44% at 4 kHz) and sex (12.09% at 8 kHz). Thus, our multivariate analysis suggests that As level in toenails is the largest contributor to hearing loss at 12 kHz among the confounders including age and smoking, while As level in toenails is the second-largest contributor after age to hearing loss at 4 and 8 kHz. On the other hand, hearing levels in males are generally known to be worse than those in females [41]. In this study, hearing thresholds in females were significantly higher than those in males. As levels in toenails (mean ± SD) in females (1.58 ± 1.00 μg/g) were significantly higher than those in males (1.17 ± 1.30 μg/g; p < 0.0001). Therefore, it is possible that there is an association between higher As levels in females and higher hearing thresholds than those in males. It would be worthwhile to investigate the reason for the gender difference in As levels.

### Possible route of exposure to As for the subjects in this study

The major route of exposure to As in the subjects in this study is not clear, but in our experimental study in which mice were orally exposed to As, there was a correlation between As levels in nails and inner ears. In our epidemiological study, we found a significant correlation (r = 0.5826; p < 0.0001) between As levels in toenails and duration of drinking tube well water (S1 Fig). Therefore, it is likely that the route of exposure to As for the subjects in this study was drinking well water. Tube well water polluted with As is known to contain other elements. In our previous studies, barium was detected at a level similar to that of As in well drinking water in Bangladesh [42] and was to be associated with hearing loss in humans [14]. In this study, significant correlations between As levels in toenails and hearing loss remained in multivariate analysis adjusted with barium in addition to the confounders (S3 Table). Thus, the results suggest that As level in toenails is independently associated with hearing loss at 4, 8 and 12 kHz in humans. Further study is needed to investigate the association between hearing loss and As levels in well water worldwide, since more than 137 million people drink tube well water polluted by As worldwide [43].

### Study limitations

This pilot study has several limitations. First, we used an ipod with headphones as the screening method for the field work in Bangladesh, since there is no clinical audiometer in rural areas. In addition, we did not perform otoscopy and tympanometry to check middle-ear problems. Second, our cross-sectional analysis was useful for determining the association between As levels in biological samples and hearing loss, but a the causal relationship could not be established. Cohort studies will be needed to determine the causality. Third, there was no information about noise background, though the subjects lived in rural areas and did not have portable music players with earphones. Fourth, the sample size of the pilot study in Bangladesh was small. Additional study is needed to analyze the association between As and hearing loss with consideration of the above limitations in larger sample sizes in other areas.

### Conclusion

In conclusion, our combined experimental study and epidemiological study showed that As levels in nails were significantly associated with hearing loss in humans and that As levels in nails were significantly associated with those in inner ears in mice. Our epidemiological study also suggests the possible thresholds of As ranging from 0.30 μg/g to 0.70 μg/g in toenails that increase the risk for hearing loss in humans. Exposure to As is a worldwide health risk for humans. Our study provides new information that As level in toenails is a reliable index to predict As-mediated hearing loss in humans living in As-polluted areas.

## Supporting information

S1 Supporting Method Determination of As levels in hair samples. (DOC)

S1 Table Adjusted ORs (95% CI) for hearing loss and As levels in hair samples (n = 145)^a^. (DOC)

S2 Table Hearing loss on McFadden’s pseudo *R*^*2*^ for each factor. (DOC)

S3 Table Adjusted ORs (95% CI) for hearing loss and As levels in biological samples (n = 145)^a^. (DOC)

S1 Fig Correlation between As levels in toenails and duration of drinking tube well water. Correlation between As levels in toenails and duration of drinking tube well water (years) was determined by spearman correlation coefficients.
(TIF)
