# Supplementary material for: Arsenic level in toenails is associated with hearing loss in humans
Source: PLoS One. 2018 Jul 5;13(7):e0198743. doi: 10.1371/journal.pone.0198743 (PMC6033376; doi:10.1371/journal.pone.0198743)
Supplement: S1 Supporting Method — (DOC) [file pone.0198743.s001.doc]

**Supporting information**

**Supporting Method**

**Determination of As levels in hair samples**

Hair samples of 1-10 cm in length were collected from the subjects. First, the hair samples were washed with detergent water and air-dried with two or three drops of acetone. Then 3 ml of HNO_3_ was added and the samples were incubated overnight at room temperature. After decomposition, the samples were incubated at 80˚C for 3 hours. The samples were further incubated in 1 ml of H_2_O_2_ at 80˚C for 3 hours. Finally, Milli-Q water was added to the samples to adjust the final volume to 5 ml. As levels in hair were measured by ICP-MS.
