# Supplementary material for: Arsenic level in toenails is associated with hearing loss in humans
Source: PLoS One. 2018 Jul 5;13(7):e0198743. doi: 10.1371/journal.pone.0198743 (PMC6033376; doi:10.1371/journal.pone.0198743)
Supplement: S1 Table — (DOC) [file pone.0198743.s002.doc]

**S1 Table. Adjusted ORs (95% CI) for hearing loss and As levels in hair samples (n = 145)^a^.**

| **As in hair** | 1 kHz  (≥ 7 dB) | 4 kHz  (≥ 10 dB) | 8 kHz  (≥ 24 dB) | 12 kHz  (≥ 45 dB) |
| --- | --- | --- | --- | --- |
| Low | Reference | Reference | Reference | Reference |
| High | 1.11  (0.44-2.79) | 1.83  (0.75-4.50) | 1.73  (0.69-4.32) | 2.94*  (1.20-7.20) |

^a^Adjusted for age, sex, smoking history and BMI. **p* < 0.05.
