# Supplementary material for: Arsenic level in toenails is associated with hearing loss in humans
Source: PLoS One. 2018 Jul 5;13(7):e0198743. doi: 10.1371/journal.pone.0198743 (PMC6033376; doi:10.1371/journal.pone.0198743)
Supplement: S2 Table — (DOC) [file pone.0198743.s003.doc]

**S2 Table. Hearing loss on McFadden’s pseudo *R^2^* for each factor.**

^a^Relative contribution of each factor was calculated using the following formula: Relative contribution [Pseudo *R^2^* (%)] = (Pseudo *R^2^*  of the final five-factor model – Pseudo *R^2^* of the nested four-factor model with the factor of interest removed)/Pseudo *R^2^* of the final five-factor model.

|  | **Relative contribution [Pseudo *R^2^* (%)]^a^** | | | |
| --- | --- | --- | --- | --- |
|  | 1 kHz | 4 kHz | 8 kHz | 12 kHz |
| Age | 15.22 | 20.75** | 20.41** | 16.53** |
| BMI | 7.21 | 3.87 | 6.64 | 0.16 |
| Sex | 0.40 | 4.86 | 12.09* | 3.75 |
| Smoking | 30.84 | 9.44* | 5.87 | 13.78* |
| As in toenails | 1.60 | 14.54** | 13.70** | 17.33** |
| Model redundancy^b^ | 44.73 | 46.54 | 41.28 | 44.44 |

^b^The remaining contribution (model redundancy) was calculated as the difference between Pseudo *R^2^*  of the final five-factor model and the sum of the relative contribution of each factors which was an estimate of the model explained by more than one factor.

p values were calculated using the logistic ratio test for each factor. *p < 0.05, **p < 0.01.
