# Supplementary material for: Arsenic level in toenails is associated with hearing loss in humans
Source: PLoS One. 2018 Jul 5;13(7):e0198743. doi: 10.1371/journal.pone.0198743 (PMC6033376; doi:10.1371/journal.pone.0198743)
Supplement: S3 Table — (DOC) [file pone.0198743.s004.doc]

**S3 Table. Adjusted ORs (95% CI) for hearing loss and As levels in biological samples (n=145)^a^.**

|  | 1 kHz  (≥ 7 dB) | 4 kHz  (≥ 10 dB) | 8 kHz  (≥ 24 dB) | 12 kHz  (≥ 45 dB) |
| --- | --- | --- | --- | --- |
| As in toenails |  |  |  |  |
| Low | Reference | Reference | Reference | Reference |
| High | 1.39  (0.46-4.25) | 4.43**  (1.54-12.73) | 3.83**  (1.42-10.29) | 4.08**  (1.51-11.03) |

OR, odds ratio; CI, confidence interval.

^a^Adjusted for age, sex, smoking history, BMI and barium levels. ***p* < 0.01.
